# Effect of Dietary Ramie Powder (*Boehmeria nivea*) at Various Levels on Growth Performance, Carcass and Meat Qualities, Biochemical Indices, and Antioxidative Capacity of *Linwu* Ducks

**DOI:** 10.3389/fphys.2022.839217

**Published:** 2022-03-09

**Authors:** Qian Lin, Yang Liu, Xin Wang, Yan-Zhou Wang, Peng Huang, Chun-Jie Liu, Li-Ping Liao, Ying-Hui Li, Zhi-Yong Fan, Jian-Guo Zeng, Si-Yuan Zhu, Hua-Jiao Qiu

**Affiliations:** ^1^Institute of Bast Fiber Crops, Chinese Academy of Agricultural Sciences, Changsha, China; ^2^College of Horticulture, Hunan Agricultural University, Changsha, China; ^3^Hunan Deren Husbandry Technology Co., Ltd., Changde, China; ^4^College of Animal Science and Technology, Hunan Agricultural University, Changsha, China

**Keywords:** ramie, growth performance, meat quality, antioxidative capacity, *Linwu* duck

## Abstract

Current experiment was designed to check the effect of dietary supplementation of ramie powder on the growth performance, carcass and meat qualities and antioxidative capacity of *Linwu* ducks. A total of 312 ducks at 21-day-age were equally divided into 4 groups, fed with control diet, control diet supplemented of 3, 6, or 12% ramie powder, respectively. The results showed that dietary supplementation of 6 and 12% ramie powder increased the final weight and daily body weight gain (*P* < 0.05), and dietary supplementation of 6% ramie improved the cooking loss of the leg meat 45-mins-postmortem compared with the control group (*P* < 0.05). Moreover, dietary supplementation of 6% ramie powder promoted the antioxidative capacity of the ducks by increasing the serum activities of superoxide dismutase and glutathione (*P* < 0.05), as well as the mRNA expressions of glutathione peroxidase 1 in the breast meat and superoxide dismutase 1 in the leg meat (*P* < 0.05). This experiment demonstrated that dietary supplementation of ramie powder showed beneficial efficacy on the growth performance of *Linwu* ducks. It corroborated the potential of dietary ramie being used as poultry feed ingredient and suggested that 6% was the proper supplementation rate of ramie powder in *Linwu* ducks’ feed.

## Introduction

In recent years, shortages of feed resources and rising prices restricted the development of animal husbandry. On the other hand, the demand of poultry meat with high quality grew in recent years, which encouraged the poultry industry consistently supplying healthy, safe and tasty poultry meat (Roenigk, 1999). Accordingly, it was imperative to look for new feed ingredients, better with beneficial effect on the meat quality of poultry, to overcome the feed shortage problems.

Ramie (*Boehmeria nivea*), well known as “China grass,” is a perennial plant of the *Boehmeria* genus under the Nettle, or *Urticaceae* family, order *Urticales* and class *Magnoliopsida* (Luan et al., 2018). It drew great attention as a type of unconventional feed source for livestock and poultry recently for its relatively low fiber content and high crude protein (about 20.00% of dry matter, DM), amino acids (especially the lysine, slightly > 1.00% of DM), minerals (especially the calcium, about 4.00% of DM) and carotene content in leaves and tender tops (Kipriotis et al., 2015). Ramie contained many biologically active compounds in its roots and leaves, such as flavonoids (Rutin, Rhoifolin, and beta-ionone) and polyphenols compounds (chlorogenic acid, ferulic acid, and caffeic acid) (Wang et al., 2019). Thus, it was practical to evaluate the effects of ramie as a new feed ingredients to the livestock. *Linwu* duck was a major duck breed in China which had strong adaptability to stressful environments (Lin et al., 2016). Therefore, *Linwu* duck was selected as an animal model for the evaluation of ramie powder as a feed ingredient in the present study.

It was commonly known that meat quality was formed by a complex interplay of various factors. The protein degradation in meat was primary caused by degradation effect of various proteases (Kemp and Parr, 2012), and the pH decreasing in meat was mainly because of the metabolism of glycogen in the muscles into lactic acid after the animal’s death (Bendall, 1979). Oxidation also partially contributed to the degradation of meat lipids and proteins, and caused the decrease of the pH values and the water holding capacity in meats, which deteriorated the tenderness, flavor, juiciness, and color of meats (Ripoll et al., 2013; Qiao et al., 2017). Previous literatures indicated that ramie leaves had a number of flavonoids and polyphenols which exerted antioxidative activities *in vitro* and cellular (Chen et al., 2014). However, to our knowledge, no relevant research was conducted to test the influence of ramie treatment on ducks’ meat quality.

The objective of the present study was to evaluate the effects of varying levels of dietary ramie powder on the growth performance of *Linwu* ducks. Additionally, we tried to verify the hypothesis that ramie treatment could increase the antioxidant status and improve the meat quality of *Linwu* ducks, by examining the activities of antioxidative enzymes and the mRNA expressions of the antioxidative enzymes, as well as the indices associated with the meat qualities.

## Materials and Methods

All the experimental procedures were conducted in accordance with the Chinese guidelines for animal welfare and approved by the Animal Care and Use Committee, Institute of Bast Fiber Crops, Chinese Academy of Agricultural Sciences.

### Preparation of Ramie Powder

The leaves and tender tops of fresh ramie (*Boehmeria nivea cv.* Qingsizhu No. 1) were cut at about 80 cm height and immediately dried at 60°C for 4 days in a heat drier room. The weight ratio of leaf to stem in dry ramie was 3.37. Then, the dried stems and leaves were crushed to powder using a grinder equipped with a 1.5 mm sieve, and kept in a well-closed and light-resistant room.

### Birds, Diets, and Experimental Design

Three hundred and twelve 21-day-old female *Linwu* ducks, free of infectious disease, were obtained from Hunan Shunhua Duck Industrial Development Company, and transferred to the laboratory of the Institute of Bast Fiber Crops, Chinese Academy of Agricultural Sciences. The ducks were supplied *ad libitum* access to feed and water throughout the trial period. After a 1-week adaptation period fed with the control diet, all the ducks were individually weighed and equally divided into 4 groups, meeting the purpose that the average initial weights among groups were not significant different. The ducks in each group (78 *Linwu* ducks) were further subdivided into six 120 cm × 120 cm cages (13 ducks/cage). Group 1 received the control diet which was based on corn, soybean meal, wheat bran, wheat flour, and rice husk. Group 2, 3, and 4 received a control diet in which corn, soybean meal, wheat bran, wheat flour, and rice husk were partly replaced by ramie powder to reach proportions of 3, 6, or 12%, respectively. All diets were formulated with similar levels of nutrient and to meet recommendations of Nutrient Requirements of Meat-type Duck (China, NY/T 2122-2012), as shown in Table 1. The feeding period was 42 days.

**TABLE 1:** Composition and nutrient levels of experimental diets (air-dry basis, %).

Items	Diets			
	0.00%	3.00%	6.00%	12.00%
**Ingredients**				
Corn	45.98	44.11	46.76	46.87
Soybean meal	25.58	24.64	24.43	23.22
Wheat flour	10.00	10.00	10.00	10.00
Rice husk	5.42	3.73	2.77	0.00
Ramie powder	0.00	3.00	6.00	12.00
Oil	2.57	3.30	2.65	2.94
Wheat bran	6.35	7.39	3.78	1.84
Limestone	1.37	1.12	0.78	0.21
CaHPO_4_⋅2H_2_O	1.27	1.24	1.33	1.40
78.5% *L*- Lys	0.00	0.01	0.03	0.05
98.5% *DL*- Met	0.16	0.16	0.17	0.17
Salt	0.30	0.30	0.30	0.30
1% Premix^1^	1.00	1.00	1.00	1.00
Total	100.00	100.00	100.00	100.00
**Nutrient levels^2^**				
Metabolizable energy, MJ/kg	11.92	11.92	11.92	11.92
Crude protein	17.00	17.02	17.01	16.99
Crude fiber	5.55	5.54	5.56	5.57
Calcium	0.85	0.83	0.84	0.86
Total phosphorus	0.59	0.59	0.57	0.58
Available phosphorus	0.35	0.35	0.36	0.36
Lysine	0.91	0.90	0.89	0.91
Methionine	0.43	0.45	0.44	0.43

*^1^The premix provided the following (per kilogram of complete diet) micronutrients: VA 12 000 IU, VD_3_ 2 500 IU, VE 20 mg, VK_3_ 3 mg, VB_1_ 3 mg, VB_2_ 8 mg, VB_6_ 7 mg, VB_12_ 0.03 mg, D-pantothenic acid 20 mg, nicotinic acid 50 mg, biotin 0.1 mg, folic acid 1.5 mg, Cu (as copper sulfate) 9 mg, Zn (as zinc sulfate) 110 mg, Fe (as ferrous sulfate) 100 mg, Mn (as manganese sulfate) 100 mg, Se (as sodium selenite) 0.16 mg, I (as potassium iodide) 0.6 mg.*

*^2^The contents of total energy, crude protein, crude fiber, calcium, total phosphorus, lysine, and methionine were analyzed.*

### Record of Growth Performance

Body weight of *Linwu* duck was individually measured at the beginning (28-day-old) and the end of the trial (70-day-old). Feed intake per cage was recorded daily. The average daily feed intake (ADFI), average daily body weight gain (ADG), and feed/gain ratios (F/G) were calculated for each cage.

### Carcass Characteristics and Sample Collection

On the last day of the trial, twelve ducks in each group (two ducks in each cage) with body weights close to the mean were selected after 12-h fasting. Blood samples were collected *via* wing vein with 10-mL heparin-free tubes, and centrifuged at 3,000 × g for 15 min at 4°C. The obtained serum samples were stored at −20°C until analysis. The ducks were then immediately slaughtered by cervical dislocation. The carcasses were bled by hanging for 5 min and scalded in water (65°C) for approximately 1-min for feathers plucking. The weights of carcasses were recorded as dressed weight. After carefully excising the gastrointestinal tract and organs, the weights of half-eviscerated, eviscerated, abdominal fat, leg muscle, breast muscle, and lean meat (total legs and breasts muscle) were recorded. Then, a 1-cm-thick sample of leg muscle and breast muscle was rapidly taken from each duck and frozen in liquid nitrogen and stored at −80°C until RNA extraction. Finally, the dressed percentage (PD), percentage of half-eviscerated yield (PHEY), percentage of eviscerated yield (PEY), percentage of breast muscle (PB), percentage of leg muscle (PL), percentage of lean meat (PLM), and percentage of abdominal fat (PAF) were calculated, respectively, according to the regulations and requirements of Performance Ferms and Measurement for Poultry (China, NY/T 823-2004).

### Meat Quality Analysis

The meat qualities of leg and breast muscles were measured as follow: the meat colors (45-min and 24-h postmortem) were determined as the L*, a*, and b*, which were the indicators of lightness, redness and yellowness, respectively, with a colorimeter (CR-400; Minolta Camera Co., Osaka, Japan). The pH values (45-min and 24-h postmortem) were measured with a pH meter (Model 340, Mettler-Toledo GmbH, Schwerzenbach, Switzerland) by the method described previously (Choi et al., 2016). The drip losses at 24-h postmortem, the cooking losses at 45-min and 24-h postmortem were assayed according to the method described previously (Qiao et al., 2017), and the shear forces were further measured with the digital tenderness meter (C-LM3B, Northeast Agricultural University, Harbin, China) after measuring the cooking loss, to evaluate the tenderness of meat (Tang et al., 2009).

### Measurement of Serum Antioxidant Biomarkers

The serum levels of glutathione (GSH), malonaldehyde (MDA) and the activities of superoxide dismutase (SOD), catalase (CAT), glutathione peroxidase (GPX), and total antioxidant capacity (T-AOC) were determined by the commercial kits (Nanjing Jiancheng Bioengineering Institute, Nanjing, China) and an automated fluorescence instrument (Thermo Fisher Scientific, Waltham, MA, United States) following the corresponding procedures. And, the serum level of 8-hydroxy-2′-deoxyguanosine (8-OHdG) was quantified by a specific ELISA kit (Abcam, Cambridge, United Kingdom).

### Quantification of mRNA Expression by Real-Time PCR

Total RNA from the leg muscle and breast muscle were isolated using Trizol reagent (TaKaRa, Tokyo, Japan), and then treated with DNase I (Thermo Fisher Scientific Inc., Waltham, MA, United States). The cDNA were synthesized from 1 μg of RNA with a RevertAid First Strand cDNA Synthesis Kit (Thermo Fisher Scientific Inc., Waltham, MA, United States) according to the manufacturer’s instructions. Based on the cloned complete sequences^1^ of nuclear factor erythroid-2-related factor 2/erythroid-derived CNC homology factor (*Nrf2/ECH*), catalase (*CAT*), superoxide dismutase-1 (*SOD1*), glutathione peroxidase-1 (*GPX1*), and β-actin from *Anas platyrhynchos*, primer pairs were designed with Primer 5.0 for quantitative real-time PCR (Table 2). The β-actin gene was used as the housekeeping gene. All primers were synthesized and purified by Sangon Biotech Co. Ltd. (Shanghai, China). Reaction volume of 20 μL mixture contained 10 μL Power SYBR Green PCR Master Mix (Applied Biosystems, Foster City, CA, United States), 1 μL cDNA template, 1 μL of the upstream and downstream primers for each targeting gene, and 7 μL sterilized deionized water. The amplification parameters for all the genes of the thermocycler (CFX Connect, Bio-Rad, Inc., Hercules, CA, United States) were a preheat period of 3 min at 95°C followed by 45 cycles of 95°C for 10 s and 55°C for 20 s, and a melting curve ramping from 65 to 95°C with an increasing temperature of 0.5°C. All sample analyses were carried out in triplicate and the average values were indexed. The target gene expressions were normalized to that of the selected reference gene, and the relative gene expressions were calculated using 2^–ΔΔ*Ct*^ method (Livak and Schmittgen, 2001). The mRNA levels were expressed as the fold change relative to the mean values of the control group, which was arbitrarily defined as 1.0.

**TABLE 2:** Primer sequences used for real-time quantitative PCR.

Primer name^1^	Sequences of the primer pair	GenBank accession NO.	Fragment length, bp
β-*Actin* sense	5′-AGTACCCCATTGAACACGGT-3′	EF667345	197
β-*Actin* antisense	5′-ATACATGGCTGGGGTGTTGA-3′		
*GPX1* sense	5′-TTCGAGAAGTGCGAGGTGAA-3′	KU048803	156
*GPX1* antisense	5′-GTTCCAGGAGATGTCGTTGC-3′		
*CAT* sense	5′-AATGTGCGTGACTGACAACC-3′	KU048802	196
*CAT* antisense	5′-ACGTTCATCCTCCTTCAGCA-3′		
*SOD1* sense	5′-TGGACCAAAGGATGCAGAGA-3′	KU048808	200
*SOD1* antisense	5′-CATTCCCAGTTAGCGTGCTC-3′		
*Nrf2/ECH* sense	5′-CGCCTTGAAGCTCATCTCAC-3′	KM109969	176
*Nrf2/ECH* antisense	5′-TTCTTGCCTCTCCTGCGTAT-3′		

*^1^GPX1, glutathione peroxidase-1; CAT, catalase; SOD1, superoxide dismutase-1; Nrf2/ECH, nuclear factor erythroid-2-related factor 2/erythroid-derived CNC homology factor.*

### Statistical Analysis

Statistical analysis of data was conducted using Statistical Package for the Social Sciences 19.0 (IBM, Armonk, NY, United States). One-way ANOVA model was performed in the present research. Replicate was used as the experimental unit (As for the results of the biomarker analysis, it means that the results of two birds in each cage are summarized as an average value). Results were presented as means and pooled standard errors of the means (SEM). Differences among means of all groups were considered significant at *P* < 0.05. The *P* values between 0.05 and 0.10 were considered as a trend. Duncan’s multiple range were further used when the main effect was significant. And the orthogonal polynomial contrasts were used to determine linear and quadratic responses of measured parameters of *Linwu* ducks to ramie powder levels.

## Results

### Growth Performance

Growth performances of ducks in different treatment groups were shown in Table 3. In comparison with the control group, the groups fed diets supplemented with ramie level at 6 and 12% had a significant increase in the average final weight, and ADG (*P* < 0.05) and had a trend in increase the ADFI (*P* = 0.063). Moreover, the greatest average final weight, ADG, and ADFI were achieved for ducks fed with the 6% ramie supplemented diet. Positive linear relationships were found between ramie dose and the average final weight (*P* = 0.009), ADG (*P* = 0.006), and ADFI (*P* = 0.013). However, there were no significant differences (*P* > 0.05) in F/G ratio among groups.

**TABLE 3:** Effect of varying ramie powder levels in diet on growth performance of *Linwu* ducks.

Item^1^	Ramie powder levels				*SEM*	*P*-value	Linear and quadratic effects of ramie powder	
							*P*-value	
	0.00%	3.00%	6.00%	12.00%			Linear	Quadratic
Average initial weight/(g)	685.897	684.615	687.180	683.333	1.218	0.736	0.655	0.618
Average final weight/(g)	1,569.662^b^	1,599.508^ab^	1,632.352^a^	1,617.897^a^	8.060	0.025	0.009	0.123
ADG/(g)	21.042^b^	21.783^ab^	22.504^a^	22.252^a^	0.188	0.021	0.006	0.134
ADFI/(g)	125.618	129.968	135.312	134.671	1.493	0.063	0.013	0.363
F/G	5.969	5.968	6.010	6.050	0.030	0.762	0.321	0.748

*^a,b^With in a row, values with different superscript letters mean significant difference (P < 0.05).*

*^1^ADG, average daily body weight gain; ADFI, average daily feed intake; F/G, feed/gain ratios.*

### Carcass Characteristics

Carcass characteristics were shown in Table 4. There were no significant differences (*P* > 0.05) in carcass characteristics among groups.

**TABLE 4:** Effect of different ramie powder levels in diet on carcass characteristics of 70 days *Linwu* ducks.

Item	Ramie powder levels				SEM	*P*-value	Linear and quadratic effects of ramie powder	
							*P*-value	
	0.00%	3.00%	6.00%	12.00%			Linear	Quadratic
Dressed percentage	84.729	86.153	85.530	84.588	0.769	0.894	0.887	0.475
Percentage of half-eviscerated yield	89.179	91.688	90.376	92.399	0.567	0.192	0.100	0.825
Percentage of eviscerated yield	78.979	81.224	80.108	82.357	0.640	0.288	0.123	0.999
Percentage of breast muscle	11.567	13.034	11.971	12.784	0.252	0.130	0.234	0.496
Percentage of leg muscle	9.979	9.713	9.769	9.832	0.234	0.984	0.866	0.745
Percentage of lean meat	21.546	22.746	21.741	22.616	0.369	0.596	0.522	0.832
Percentage of abdominal fat	1.475	1.281	1.393	1.281	0.072	0.760	0.494	0.789

### Meat Quality

Meat qualities of the *Linwu* ducks were shown in Tables 5, 6. In breast muscles, no effect of dietary ramie supplementation was detected on the shear force, 24 h pH value, 45 min and 24 h meat color or cooking loss. However, there were trends in increase in the pH value at 45 min (*P* = 0.081) and decrease in the drip loss (*P* = 0.096) in the groups treated with ramie. In addition, quadratic effects (*P* < 0.05) on drip loss and pH value at 45 min were observed as the dietary ramie level increased from 0 to 12%, and the lowest drip loss value and the highest 45 min pH value were noted, respectively, in ducks fed with 6 and 3% ramie.

**TABLE 5:** Effect of different ramie powder levels in diet on meat quality of 70 days *Linwu* ducks’ breast muscle.

Item^1^		Times	Ramie powder levels				*SEM*	*P*-value	Linear and quadratic effects of ramie powder	
									*P*-value	
			0.00%	3.00%	6.00%	12.00%			Linear	Quadratic
Shear force, kg⋅f		45 min	3.229	3.080	3.227	3.398	0.205	0.965	0.741	0.718
		24 h	3.169	3.121	3.282	2.965	0.155	0.922	0.762	0.687
Meat color	L*	45 min	34.663	34.073	35.253	33.772	0.566	0.821	0.781	0.711
		24 h	34.982	34.212	34.332	35.668	0.647	0.865	0.724	0.448
	a*	45 min	17.840	17.558	18.313	16.507	0.500	0.654	0.490	0.468
		24 h	20.643	19.805	20.362	18.570	0.538	0.563	0.262	0.669
	b*	45 min	4.127	4.833	4.525	4.982	0.206	0.495	0.239	0.767
		24 h	4.405	4.040	4.183	4.613	0.189	0.747	0.666	0.324
Cooking loss, %		45 min	38.800	37.002	37.208	37.925	0.542	0.668	0.634	0.274
		24 h	40.661	38.914	38.764	37.973	0.603	0.474	0.147	0.699
Drip loss, %		24 h	3.230	2.653	2.346	2.860	0.130	0.096	0.199	0.033
pH value		45 min	6.291	6.403	6.381	6.308	0.019	0.081	0.848	0.013
		24 h	6.142	6.190	6.169	6.202	0.023	0.824	0.466	0.868

^1^L*, lightness; a*, redness; b*, yellowness.

**TABLE 6:** Effect of different ramie powder levels in diet on meat quality of 70 days *Linwu* ducks’ leg muscle.

Item^1^		Times	Ramie powder levels,				*SEM*	*P*-value	Linear and quadratic effects of ramie powder	
									*P*-value	
			0.00%	3.00%	6.00%	12.00%			Linear	Quadratic
Shear force, kg⋅f		45 min	3.430	2.761	3.302	3.399	0.143	0.328	0.727	0.191
		24 h	3.820	3.326	3.391	3.238	0.166	0.638	0.283	0.623
Meat color	L*	45 min	37.610	38.475	37.812	36.447	0.685	0.791	0.524	0.446
		24 h	36.638	36.405	37.305	33.893	0.605	0.209	0.174	0.187
	a*	45 min	19.267	18.518	19.808	21.623	0.629	0.363	0.150	0.317
		24 h	19.938	18.190	19.885	20.405	0.470	0.380	0.468	0.239
	b*	45 min	5.393	5.745	6.025	5.735	0.296	0.915	0.647	0.614
		24 h	4.365	4.408	4.988	5.530	0.317	0.548	0.174	0.704
Cooking loss, %		45 min	31.954^a^	30.035^ab^	27.178^b^	32.647^a^	0.771	0.043	0.899	0.013
		24 h	31.867	30.751	30.523	29.564	0.604	0.636	0.214	0.950
Drip loss, %		24 h	2.902	2.798	2.378	2.573	0.123	0.463	0.220	0.553
pH value		45 min	6.347^ab^	6.370^ab^	6.453^a^	6.301^b^	0.020	0.048	0.728	0.025
		24 h	6.162	6.272	6.245	6.214	0.018	0.159	0.404	0.051

*^a,b^With in a row, values with different superscript letters mean significant difference (P < 0.05).*

^1^L*, lightness; a*, redness; b*, yellowness.

In leg muscles, compared with the control group and 12% ramie treated group, the 6% ramie treated group had a significant decrease in the cooking loss at 45 min (*P* = 0.043). Compared with the 12% ramie treated group, the 6% ramie treated group had a significant increase in the pH value at 45 min (*P* = 0.048). Quadratic effects (*P* < 0.05) for 45 min cooking loss and pH value were observed as the dietary ramie level increased from 0 to 12%. However, there were no significant differences (*P* > 0.05) on other characteristics of leg muscles quality among groups.

### Serum Antioxidant Biomarkers

Levels of serum antioxidant biomarkers were shown in Table 7. In comparison with the control group, the groups supplemented with 6% ramie had a significant increase in the content of GSH and the activity of SOD (*P* < 0.05). In addition, there were increasing trends in the levels of GPX (*P* = 0.082) and T-AOC (*P* = 0.092) in the groups supplemented with ramie. Moreover, positive linear relationships were observed between ramie dose and serum levels of GPX and GSH (*P* < 0.05). Besides, significant quadratic relationships were observed between ramie dose and serum levels of SOD, and T-AOC (*P* < 0.05).

**TABLE 7:** Effect of different ramie powder levels in diet on serum antioxidant biomarks of 70 days *Linwu* ducks.

Item^1^	Ramie powder levels				*SEM*	*P*-value	Linear and quadratic effects of ramie powder	
							*P*-value	
	0.00%	3.00%	6.00%	12.00%			Linear	Quadratic
SOD, U/ml	61.005^b^	66.378^ab^	70.628^a^	63.191^b^	1.287	0.035	0.293	0.010
GPX, U/ml	248.918	260.983	276.539	268.266	4.003	0.082	0.035	0.178
CAT, U/ml	1.056	1.144	1.305	1.233	0.048	0.290	0.113	0.401
GSH, μmol/L	25.789^b^	28.583^ab^	34.169^a^	33.020^a^	1.249	0.049	0.012	0.383
T-AOC, mmol/L	0.735	0.820	0.805	0.762	0.014	0.092	0.552	0.018
8-OHdG, ng/ml	19.114	18.598	16.707	18.919	0.643	0.556	0.677	0.310
MDA, nmol/ml	6.210	6.157	5.764	6.114	0.114	0.534	0.520	0.398

*^a,b^With in a row, values with different superscript letters mean significant difference (P < 0.05).*

*^1^SOD, superoxide dismutase; GPX, glutathione peroxidase; CAT, catalase; GSH, glutathione; T-AOC, total antioxidant capacity; 8-OHdG, 8-hydroxy-2′-deoxyguanosine; MDA, malonaldehyde.*

### The mRNA Expression Levels of Muscular Antioxidant-Related Genes

The mRNA expression levels of muscular antioxidant-related genes were shown in Table 8. In breast muscles, compared with the control group, the group with ramie level at 6% had significant increase in the *GPX1* mRNA expression level (*P* < 0.05). A quadratic effect was observed between the dietary ramie level and for *GPX1* mRNA expression level (*P* = 0.012).

**TABLE 8:** Effect of different ramie powder levels in diet on antioxidant-related genes expression in muscle of 70 days *Linwu* ducks.

Item^1^	Ramie powder levels				*SEM*	*P*-value	Linear and quadratic effects of ramie powder	
							*P*-value	
	0.00%	3.00%	6.00%	12.00%			Linear	Quadratic
**Breast muscle**								
*GPX1*	1.000^b^	1.186^ab^	1.284^a^	1.086^ab^	0.040	0.049	0.250	0.012
*SOD1*	1.000	1.092	1.186	0.975	0.066	0.709	0.975	0.301
*CAT*	1.000	1.101	1.106	1.035	0.035	0.699	0.747	0.269
*Nrf2/ECH*	1.000	1.057	1.088	1.069	0.048	0.939	0.621	0.721
**Leg muscle**								
*GPX1*	1.000	1.015	1.169	0.898	0.060	0.503	0.788	0.267
*SOD1*	1.000^b^	1.111^ab^	1.303^a^	1.068^b^	0.042	0.048	0.223	0.028
*CAT*	1.000	1.286	1.407	1.217	0.061	0.098	0.127	0.043
*Nrf2/ECH*	1.000	0.800	1.087	1.129	0.075	0.451	0.336	0.438

*^a,b^With in a row, values with different superscript letters mean significant difference (P < 0.05).*

*^1^GPX1, glutathione peroxidase-1; SOD1, superoxide dismutase-1; CAT, catalase; Nrf2/ECH, nuclear factor erythroid-2-related factor 2/erythroid-derived CNC homology factor.*

In leg muscles, compared with the control group and the 12% ramie treated group, the 6% ramie treated group showed a significant increase in the *SOD1* mRNA expression level (*P* = 0.048). There was an increasing trend in the *CAT* mRNA expression level in the ramie treated groups (*P* = 0.098). In addition, quadratic effects were observed between dietary ramie level and *SOD1* and *CAT* mRNA expression level (*P* < 0.05). However, dietary ramie supplementation did not influence the mRNA expression levels of *Nrf2/ECH* in either breast muscles or leg muscles of the ducks.

## Discussion

Feed shortage is one of the most critical factors to impede the development of animal husbandry in many regions, which is in particular severe in southern China (Yin et al., 2019). Ramie, a hardy perennial herbaceous plant with high production of DM, is widely grown in southern China (Lv, 2012). Furthermore, the nutritive value of ramie is reported to be similar to that of Lucerne (Kipriotis et al., 2015). We selected *Linwu* duck in the current research because it was a famous and excellent breed of duck in local southern China with outstanding meat quality and strong adaptability. Therefore, this research was important and practical in assisting the *Linwu* duck breeding industry with recommendation of utilization of a new feed ingredient.

In the present study, the effects of ramie powder as feed sources in the growth performance and carcass characteristics of *Linwu* ducks were tested. Among the diet composition of all groups, no differences in nutrition or ingredient levels in diets were designed, except for the content of corn, soybean meal, rice husk, and ramie powder. Ramie powder as a supplementation to traditional feed material was considered as a provider of crude protein and crude fiber in the diets. Previous study showed that, 9 and 12% inclusion of ramie in diet lowered the final body weight and ADG of the finishing pigs compared to the ones with control diets, but the ADFI did not changed (Li et al., 2019). It was partially different with our finding, that 6 and 12% of ramie supplement in diets significantly increased the final body weight and ADG of *Linwu* ducks, but no influence was found in ADFI by ramie inclusion. The possible explanation for the inconsistence could be the difference in dietary crude fiber level of the diet. It was reported that excessive intake of fiber could reduce the nutrient digestibility and increase the satiety of birds, leading to the reduction of weight gain (Nielsen et al., 2011). In the present study, the diets in all experimental groups possessed similar crude fiber contents, while in Li’s study, the crude fiber level was raised as ramie inclusion level increased in diet.

On the other hand, no differences were noticed with the parameters of the carcass characteristics of *Linwu* ducks among groups, indicating that ramie supplementation in diets would not harm the productive efficacy of the ducks compared with the control diets. Duck meat was famous worldwide, especially in Asia, due to its attractive flavor, taste, and nutritious value (Chen et al., 2010). Increasing demands for high-quality meat called for new feed formula and animal breeding for better meat quality (Mehta et al., 2015). The tenderness, flavor, juiciness and color were commonly accepted as the main elements associated with the meat quality (Qiao et al., 2017). In current research, the quality of breast and leg muscles of ducks in different groups were studied. It was found that 6% ramie supplementation in diets significantly decreased the cooking loss of leg muscle at 45 min post-slaughter, indicating that ramie powder would improve the water holding capacity and improved the juiciness of the duck meat. The result was partially corroborated by Li’s study, that finishing pigs fed diets supplemented with less than 9% ramie demonstrated an improvement in the pork quality (Li et al., 2019). Moreover, the inclusion of ramie hay or raw ramie in diets showed a tendency for better meat quality in goats as well (Zhang et al., 2019).

Oxidation stress was mainly caused by overproduction of reactive oxygen species (ROS), which contributed to the biological damages that affect the growth and production in animals (Nisar et al., 2013). It also led to lipid peroxidation that deteriorated the meat tenderness by inhibiting calpain activity and suppressing proteolysis process (Harris et al., 2001). In organism and muscle tissue, the balance between the oxidants and antioxidants was modulated by a defense system, composed by enzymatic components such as SOD, CAT, and GPX, and non-enzymatic compounds such as vitamin C and GSH (Surai et al., 2019). Studies showed that dietary supplements containing natural antioxidant plant materials could promote the growth of poultry, improved the antioxidant defense system of heat stressed birds, and improved meat and egg quality (Abo Ghanima et al., 2019; Ashour et al., 2020; Dosoky et al., 2021). In poultry, the antioxidant defense system was mediated by Nrf2/ECH, which triggered the antioxidant response elements and promote the expression of antioxidative enzymes in various tissues (Nguyen et al., 2009). When oxidative damage happened, MDA and 8-OHdG were formed as predominant forms of ROS induced oxidative lesions, so that they were considered common biomarkers for oxidative stress (Valavanidis et al., 2009; Fu et al., 2013). In order to prevent the oxidative stress, dietary ingredients that promoted the activities of antioxidant defense system were widely used in animal feed (Akbarian et al., 2016). At present, there was growing interests in utilizing herbal medicines as supplementation in feed for poultry and livestock to maintain or improve their health and productivity. The benefits to host health were mainly attributed to the phytochemical metabolites, namely polyphenols. Polyphenols were natural antioxidants with effects as antioxidant, immune regulation, antibacterial, antiviral, detoxification, and prevention of liver toxicity (Abd El-Hack et al., 2019; Abdel-Moneim et al., 2020; Attia et al., 2020; Mesalam et al., 2021). Previous data showed that ramie leaf contained polyphenols and flavonoids which were considered excellent antioxidant (Lee et al., 2014). They could effectively activate the antioxidant enzymes in intestinal mucosa (Lee et al., 2020) and muscle tissue (Li et al., 2019). In the current study, 6 and 12% ramie supplementation in diets increased the serum activity of SOD and content of GSH compared to the control group. Moreover, increased expressions of SOD1 in leg muscle and GPX1 in breast muscle were noticed under the dietary supplementation of 6% ramie. Both results above were in lines with previous findings and confirmed the antioxidative efficacy of ramie.

In conclusion, this study suggested a dietary supplementation of 6% ramie powder could promote the growth performance of *Linwu* ducks with no adverse effect on meat quality, possibly due to improvements of the antioxidative capabilities in ducks. This study provided solid information for the utilization of ramie as feed source for poultry.

## Data Availability Statement

The original contributions presented in the study are included in the article/supplementary material, further inquiries can be directed to the corresponding authors.

## Ethics Statement

The animal study was reviewed and approved by the Animal Care and Use Committee, Institute of Bast Fiber Crops, Chinese Academy of Agricultural Sciences.

## Author Contributions

All authors participated in the discussion, edited the manuscript, and approved the submitted version.

## Conflict of Interest

QL and YL were employed in Hunan Deren Husbandry Technology Co., Ltd. The remaining authors declare that the research was conducted in the absence of any commercial or financial relationships that could be construed as a potential conflict of interest.

## Publisher’s Note

All claims expressed in this article are solely those of the authors and do not necessarily represent those of their affiliated organizations, or those of the publisher, the editors and the reviewers. Any product that may be evaluated in this article, or claim that may be made by its manufacturer, is not guaranteed or endorsed by the publisher.

## Footnotes

1
https://www.ncbi.nlm.nih.gov/genbank/
